# Athlete monitoring in handball (ATHMON HB): a systematic review protocol

**DOI:** 10.1186/s13643-025-02806-2

**Published:** 2025-03-15

**Authors:** Alexander-Stephan Henze, Johannes Kirsten, Lynn Matits, Daniel Alexander Bizjak, Sebastian Viktor Waldemar Schulz

**Affiliations:** 1 Sports and Rehabilitation Medicine, University Hospital Ulm, Leimgrubenweg 14, 89075 Ulm, Germany; 2 Clinical & Biological Psychology, Institute of Psychology and Education, Ulm University, Albert-Einstein-Allee 47, 89081 Ulm, Germany

**Keywords:** Load management, Team sports, Athletic performance, Heart rate determination, Biomarkers, Athlete self-reported measures, Postexercise recovery

## Abstract

**Background:**

Athlete monitoring has become an important aspect of demanding indoor team sports like handball. Monitoring external and internal load and well-being should help coaches to optimize load management and recovery strategies to improve athletic performance and maintain athlete health. However, there is a large variety in the methods described, their coherence, and their effect on performance and health. This systematic review will summarize the methods currently used to monitor external and internal load and well-being during training and competition in team handball.

**Methods:**

This systematic review will follow the current Preferred Reporting Items for Systematic reviews and Meta-Analyses (PRISMA) guidelines. A comprehensive search of electronic databases, including MEDLINE, Embase, and Web of Science, will be conducted until 30 April 2024. Original articles in English, German, or Spanish that assess methods for monitoring the external and/or internal load and/or well-being of healthy team handball players during training and/or competition will be considered. In addition, a backward search of all relevant full-text articles identified through the search will be conducted. Two reviewers will independently perform study selection, data extraction, and risk-of-bias assessment for included studies. Included studies will be assessed for risk of bias using the Newcastle–Ottawa scale. It is anticipated that the number of eligible studies will be limited, and that the data extracted from them will be highly heterogeneous. Consequently, a narrative synthesis of the extracted data will be presented.

**Discussion:**

This systematic review will provide the current evidence on methods for monitoring external and internal load and well-being in competitive team handball players. The results may assist practitioners in implementing an athlete monitoring. We will share our findings through scientific conferences, educational meetings for coaches and medical staff, expert meetings, and publication in a peer-reviewed journal.

**Systematic review registration:**

PROSPERO CRD42024540676.

**Supplementary Information:**

The online version contains supplementary material available at 10.1186/s13643-025-02806-2.

## Background

### Important characteristics and relevance

Elite team handball places great physical, mental, and emotional demands on the players. In addition, the increasing number of competitions puts players at risk of injury and other health problems. Athlete monitoring is therefore becoming a key factor in the daily fine-tuning of training load management and recovery strategies to enhance/maintain performance and reduce the risk of injury [1–5]. In recent years, technological advances have led to new methods for monitoring the external load in indoor team sports athletes [6, 7]. Local positioning system (LPS) or inertial measurement unit (IMU)-based tracking systems [8–14] have replaced time-motion analysis (TMA) [15–24] in team handball to accurately assess the physical demands associated with different playing positions during training and match play. In addition, these tracking systems are able to measure various external load and performance parameters in real time [8–14]. An external load causes a different degree of internal load at different functional levels (e.g., musculoskeletal system, cardiorespiratory system, metabolism, autonomic nervous system) [2, 5]. Therefore, various objective and subjective (surrogate) markers have been used in science and practice for internal load monitoring. These include heart rate-based measures [11, 12, 15, 18, 25, 26], biomarkers [27–30], motor test parameters, and subjective methods based on the rating of perceived exertion (RPE) [10, 14, 31, 32]. Athlete self-report measures are also used to assess the current well-being, which indicates how the athlete is coping with the load [14, 30, 31]. In general, a sport-specific multimodal approach using a combination of objective and subjective external and internal load and well-being parameters is recommended, rather than a single instrument, to provide the most comprehensive picture of the player’s current status [33]. Consequently, the selection of appropriate methods should consider the physical and mental demands of the respective sport, the specific environment, and the available resources [34].

There is currently no systematic overview of athlete monitoring in team handball, nor.

are there clear recommendations for the selection of methods for a useful multimodal monitoring approach.

### Rationale and justification

The rationale of this systematic review will be to evaluate the methodological approaches used to monitor the external [8–24] and internal load [10–12, 14, 15, 18, 25–32] and well-being [14, 30, 31] of team handball players in their daily routine, which have been investigated in observational studies to date. This systematic review will provide an overview and critical appraisal of monitoring methods in team handball to assist practitioners in implementing athlete monitoring to reduce the risk of injury and other sport-related health problems and to improve or maintain performance.

### Specification and objectives

This systematic review will summarize the current evidence and provide a critical evaluation of the methods used to monitor external and internal load and well-being in team handball. To this end, the following research question will be addressed using the PICOS (participants, interventions, comparators, outcomes, study designs) framework: What methods and parameters are currently used to monitor the external and/or internal load and/or well-being of competitive team handball players during training and/or competition?

## Methods/design

This systematic review will be conducted and reported according to the Preferred Reporting Items for Systematic reviews and Meta-Analyses (PRISMA) 2020 statement [35]. This protocol has been registered in the International Prospective Register of Systematic Reviews (PROSPERO, CRD42024540676) and adheres to the PRISMA extension for protocols (PRISMA-P) guidance (see additional file 1) [36].

### Eligibility criteria

The eligibility criteria for studies in this systematic review are adapted to the PICOS acronym, detailed as follows:

#### Participants

The present systematic review will consider studies of healthy female or male team handball players, who are engaged in competitive play within an organized structure.

#### Intervention(s)

Studies involving methods to monitor external and/or internal load and/or well-being during training and/or competition will be included.

#### Control

Studies may include other athletes or nonathletes as a control group.

#### Outcomes

The main outcomes will include external and/or internal load parameters, surrogate markers of internal load, and well-being measures. Additional outcomes will include parameters of athletic performance, injuries, or other sport-related health problems.

#### Study designs

Original observational studies (i.e., retrospective and prospective cohorts, case–control studies) will be included, if the article is written in English, German, or Spanish and published in a peer-reviewed journal. Reviews, editorials, or reports will be excluded if they do not present original data on the above interventions and outcomes of interest.

### Information sources

Three electronic databases (MEDLINE via PubMed, Embase via Ovid, Web of Science) will be systematically searched on 30 April 2024. Reference lists of identified full-text records will be manually screened using a backward search to identify potentially relevant articles not retrieved by our search strategy. The search will be repeated shortly before the final analysis to include potential any newly published articles that meet the eligibility criteria.

### Search strategy

A sensitive search strategy will be chosen to capture the evidence as comprehensively as possible. The search equation will include combinations of keywords and controlled vocabulary on the topics of team handball ("handball", "throwing sport", "overhead sport"), external and internal load and perceptual well-being ("load", "workload", "stress", "well-being", "wellness"), and monitoring methods and parameters ("monitoring", "surveillance", “measure”, "wearable electronic devices", "heart rate determination", "biomarkers", "motor tests", "jump test", "inventories", "rating of perceived exertion", "surveys and questionnaires"). Additional file 2 shows an exemplary illustration of the PubMed search equation.

### Data management

Articles from the various databases will be imported and merged into Zotero (7.0.1, George Mason University, Fairfax, VA, USA). Any duplicates will be removed either automatically or manually, and the most recent version will be retained. The list of articles will be exported to the Covidence systematic review software (Veritas Health Innovation, Melbourne, Australia) for the subsequent selection process and data extraction.

### Selection process

Two reviewers with expertise in the field of athlete monitoring in team sports will first independently screen the titles and abstracts of the identified articles against the predefined eligibility criteria. Following the screening of titles and abstracts, the full-text versions of the remaining studies will be independently screened for eligibility by the same two reviewers. After screening the full-text versions, the reference lists of the included studies will be reviewed to identify potential studies not included in the original search. If the two authors disagree about the inclusion of an article at any stage of the selection process, the matter will be discussed until a consensus is reached. If disagreement persists, a third author will be consulted to reach a consensus.

### Data collection process

Data from each article will be extracted by two independent reviewers using the data extraction tool of the Covidence systematic review software (Veritas Health Innovation, Melbourne, Australia). All authors will review the extracted data for accuracy and completeness. Should pertinent data be absent, the authors will be contacted by email and requested to provide the necessary information. If the original data will not be provided by the authors, the mean and standard deviation will be retrieved from graphical representation using WebPlotDigitizer (4.7, Austin, TX, USA). Participant characteristics, control group characteristics, and outcome variables will be reported as mean ± standard deviation (SD) where available.

### Data items

We will retrieve information on the following:

- Study characteristics (authors, year of publication, country of study population, study design, settings, study period covered)
- The characteristics of the study population and, if available, the control group (total sample size, age range, anthropometric data, competitive level, years of competitive team handball, playing position)
- The characteristics of the type of load studied (external and/or internal load, training, and/or match load)
- The characteristics of the well-being measures studied
- The characteristics of the outcomes studied (type, time points and frequency of measurement, type of effect measure, crude, and adjusted effect measures and their 95% confidence intervals).

#### Main outcomes

- ▪ External load parameters (i.e., exercise or playing duration [min], total distance covered [m], absolute number of sprints, jumps, accelerations, decelerations, changes of direction)
- ▪ Heart rate (HR)-based measures (i.e., HR exercise [beats per min], HR recovery)
- ▪ Biomarkers (i.e., creatine kinase [U/L], urea [mg/dL])
- ▪ Rating of perceived exertion (RPE) or session RPE [arbitrary units, AU]
- ▪ Motor test parameter (i.e., jump height [m] of the counter movement jump)
- ▪ Well-being measures (i.e., Likert-scale type items ranging from 0 to 6)

#### Additional outcomes

- Athletic performance parameters (i.e., maximum speed [m/s])
- Absolute or relative numbers of injuries and other sport-related health problems
- Adjustment variables

A pilot extraction phase will be conducted with three studies, after which the process will be repeated with additional studies until an acceptable level of agreement is reached. In the event of disagreement, an attempt will be made to reach a consensus between the two reviewers, or, if necessary, a third reviewer will be involved. Authors of studies with missing or unclear information will be contacted as described above. If this is unsuccessful, the data will be considered missing.

### Risk of bias in individual studies

Two independent reviewers will assess the methodological quality of all studies according to the Newcastle–Ottawa scale [37–39]. If the two reviewers will disagree about the risk of bias in an article, the matter will be discussed until a consensus will be reached. Should any further disagreement persist, a third author will be consulted in order to reach a consensus.

### Data synthesis

The complete selection process will be described using a PRISMA flowchart. It is anticipated that the number of eligible studies will be limited, and that the data extracted from them will be highly heterogeneous. Consequently, the extracted data will be synthesized in narrative form, describing the studies and PICOS elements. A graphical synthesis of the risk-of-bias assessments will be conducted for each study, according to each domain of bias and the overall risk of bias.

## Discussion

This systematic review will provide a synthesis of the current evidence on methods to monitor external and internal load and perceptual well-being in competitive team handball players during training and competition. The results may assist practitioners in implementing a comprehensive athlete monitoring. We will disseminate our findings through scientific conferences, educational meetings for coaches and medical staff, expert meetings, and publication in a peer-reviewed journal.

## Supplementary Information

Additional file 1. PRISMA-P 2015 Checklist.

Additional file 2. SEARCH STRATEGY.

## Data Availability

The datasets used and/or analyzed during the current study are available from the corresponding author upon reasonable request.
