# Supplementary material for: Athlete monitoring in handball (ATHMON HB): a systematic review protocol
Source: Syst Rev. 2025 Mar 15;14:64. doi: 10.1186/s13643-025-02806-2 (PMC11909999; doi:10.1186/s13643-025-02806-2)
Supplement: Supplementary file 2 — Additional file 2. SEARCH STRATEGY. [file 13643_2025_2806_MOESM2_ESM.docx]

**SEARCH STRATEGY**

**MEDLINE (PubMed) search strings:**

(“handball” [tw] OR “handballers” [tw] OR “throwing sports” [tw] OR “throwing sport” [tw] OR “overhead sports” [tw] OR “overhead sport” [tw])

AND

(“load” [tw] OR “loads” [tw] OR “training load” [tw] OR “workload” [MeSH Terms] OR “workload” [tw] OR “workloads” [tw] OR “stress” [tw] OR “well-being” [tw] OR “wellbeing” [tw] OR “wellness” [tw])

AND

(“monitoring” [tw] OR “monitor*” [tw] OR “monitoring, physiologic” [MesH Terms] OR “surveillance” [tw] OR “measure” [tw] OR “measure*” [tw] OR “wearable electronic devices” [MeSH Terms] OR “wearable electronic devices” [tw] OR “heart rate determination” [MeSH Terms] OR “heart rate” [tw] OR “biomarkers” [MeSH Terms] OR “biomarkers” [tw] OR “biomarker” [tw] OR “motor test*” [tw] OR “jump test*” [tw] OR “neuromuscular function” [tw] OR “surveys and questionnaires” [MeSH Terms] OR “survey” [tw] OR “questionnaire” [tw] OR “questionnaires” [tw] OR “inventory” [tw] OR “inventories” [tw] OR “rpe” [tw] OR “rating of perceived exertion” [tw])
